# Circulating levels of CD34^+^ cells predict long-term cardiovascular outcomes in patients on maintenance hemodialysis

**DOI:** 10.1371/journal.pone.0223390

**Published:** 2019-10-04

**Authors:** Ahmad Baseer Kaihan, Manabu Hishida, Takahiro Imaizumi, Masaki Okazaki, Ahmad Naseer Kaihan, Takayuki Katsuno, Akihiko Taguchi, Yoshinari Yasuda, Naotake Tsuboi, Tomoki Kosugi, Shoichi Maruyama

**Affiliations:** 1 Department of Nephrology, Graduate School of Medicine, Nagoya University, Nagoya, Japan; 2 Faculty of Medicine, Balkh University, Mazar-i-Sharif, Afghanistan; 3 Department of Epidemiology, Johns Hopkins Bloomberg School of Public Health, Baltimore, Maryland, United States of America; 4 Department of Nephrology and Rheumatology, Graduate School of Medicine, Aichi Medical University, Nagakute, Japan; 5 Department of Regenerative Medicine Research, Institute of Biomedical Research and Innovation, Kobe, Japan; 6 Department of Nephrology, Fujita Health University Graduate School of Medicine, Toyoake, Japan; International University of Health and Welfare, School of Medicine, JAPAN

## Abstract

CD34^+^ cells maintain vascular homeostasis and predict cardiovascular outcomes. We previously evaluated the association of CD34^+^ cells with cardiovascular disease (CVD) events over 23 months, but long-term CVD outcomes in relation to levels of CD34^+^ cells in patients on maintenance hemodialysis are unclear. Herein, we analyzed the long-term predictive potential levels of CD34^+^ cells for CVD outcomes and all-cause mortality. Between March 2005 and May 2005, we enrolled 215 patients on maintenance hemodialysis at Nagoya Kyoritsu Hospital and followed them up to 12.8 years. According to the CD34^+^ cell counts, patients were classified into the lowest, medium, and highest tertiles. Levels of CD34^+^ cells were analyzed in association with four-point major adverse CV events (MACEs), CVD death, and all-cause mortality. In univariate analysis age, smoking habit, lower geriatric nutrition risk index, lower calcium × phosphate product, and lower intact parathyroid hormone were significantly associated with the lowest tertile. Whereas, in multivariate analysis, age and smoking habit were significantly associated with the lowest tertile. Among 139 (64.7%) patients who died during a mean follow-up period of 8.0 years, 39 (28.1%) patients died from CVD. Patients in the lowest tertile had a significantly lower survival rate than those in the medium and highest tertiles (p ≤ 0.001). Using multivariable analyses, the lowest tertile was significantly associated with four-point MACEs (hazard ratio 1.80, p = 0.023) and CVD death (hazard ratio 2.50, p = 0.011). In conclusion, our long-term observational study revealed that a low level of CD34^+^ cells in the circulation predicts CVD outcomes among patients on maintenance hemodialysis.

## Introduction

Cardiovascular disease (CVD) is the main reason for death in patients on maintenance hemodialysis (HD)[1]. However, risk assessment for CVD cannot be fully explained using conventional risk factors including hypertension, glucose intolerance, an abnormal lipid profile, and uremia-related risk factors such as hemodynamic overload, deterioration of calcium metabolism, etc[2–5]. Therefore, development of biomarkers to improve individual risk prediction among patients receiving maintenance HD is needed.

Over more than a decade, many studies have demonstrated that endothelial progenitor cells (EPCs), in particular, circulating CD34^+^ cells, play an essential role in angiogenesis and vascular homestasis[6–11]. A reduction in EPCs including the CD34^+^ cell count may predict future CVD events in patients with coronary artery disease, type 2 diabetes, and metabolic syndrome[12–16]. Focusing on patients with end-stage renal disease, we and others have separately evaluated the significance of the circulating CD34^+^ cell count to predict CVD outcomes in patients on maintenance HD[17,18], but the association of circulating CD34^+^ cells with patient outcomes combined with CVD-related mortality was not fully demonstrated because few CVD deaths occurred during the short follow-up of 2 years[17,18]. Therefore, further studies are needed to evaluate the long-term predictive potential of CD34^+^ cells, especially in patients with a strong concern regarding CVD events such as those on maintenance dialysis.

To fill this knowledge gap, we examined the long-term association between CD34^+^ cells and CVD events in patients on maintenance HD by extending the follow-up period of our previous study (from an average of 23 months to an average of 8.0 years). We also explored whether the associations are consistent for all-cause mortality. Additionally, we assessed factors associated with low levels of CD34^+^ cells.

## Materials and methods

### Study population

We enrolled all consecutive patients who received maintenance HD at Nagoya Kyoritsu Hospital between March 2005 and May 2005. Patients with infectious diseases, malignant diseases, and a vascular event within 30 days after estimation of the number of circulating CD34^+^ cells were excluded from this study, leaving a final study sample of 215 patients.

### Study design and follow-up

This was a pseudoprospective study design[13]. All baseline data, such as demographics, dialysis vintage, and comorbidities, were recorded at the time of CD34^+^ cell estimations as reported previously[18], and follow-up data were gathered retrospectively from electronic medical records from December 2017 to baseline. The routine assessment of patients undergoing three rounds of HD and standardization of medical records in the electronic database permitted this gathering of information[13]. Patients were observed for CVD by routine screening tests including electrocardiogram and chest X-ray, which were performed every month. Echocardiography examination and a treadmill exercise tolerance test were performed annually. Patients with unusual clinical findings in routine tests or with signs and symptoms of coronary artery disease underwent angiography as described previously[18].

### Outcome definition

The outcomes of interest were four-point major adverse CV events (four-point MACEs), CVD death, and all-cause mortality. The four-point MACE was a composite of CVD-related events that included CVD death, nonfatal myocardial infarction, nonfatal stroke, and hospitalization for heart failure or unstable angina[13]. CVD death was defined as sudden death, death that occurred following acute myocardial infarction, stroke within 2–4 weeks, or any other CVD causes (e.g., valvular heart disease, arrhythmias, pulmonary embolism, or intervention)[13]. We also considered all-cause mortality as an outcome. Patients were followed until death, loss to follow-up, renal transplantation, recovery from dialysis therapy, or end of follow-up on December 2017 (i.e., administrative censoring). Previous events were determined according to the medical records and interviews with patients. Causes of death were determined by examination of hospital records, autopsy reports, and medical files of the patients’ general practitioners[18].

### Measurement of circulating CD34^+^ cells

The absolute count of CD34^+^ cells, which included endothelial lineage and immature hematopoietic stem cells in peripheral blood was measured with flow cytometry according to the International Society of Hematotherapy and Graft Engineering guidelines as reported in our prior study[18]. The reproducibility of flow cytometry method is shown in our previous paper [18] Relative CD34^+^ cell counts were determined as the ratio of circulating CD34^+^ cells to white blood cells (WBC). In this paper, we used the levels of relative CD34^+^ cells[13], which were classified into the lowest (<0.06), medium (≥0.06 and <0.09), and highest (≥0.09) tertiles according to the relative CD34^+^ cell counts.

### Statistical analyses

Data were summarized as the mean ± standard deviation (SD) or as the number (frequency). Variables with a skewed distribution were transformed to logarithmic form for analysis. The mean ± SD values of continuous variables among tertiles were compared with a one-way analysis of variance test, and categorical variables were assessed with the Kruskal-Wallis H test. Logistic regression analysis was conducted to examine the relationship between the lowest tertile as a dependent variable and each of clinical parameters, biochemical markers, and medication as an independent variable. The goodness of fit of multivariate model was tested by Hosmer-Lemeshow test. Survival curves were plotted with the Kaplan-Meier method, and the differences in survival rates among tertiles were evaluated with the Log-rank test. Furthermore, a restricted cubic spline curve was plotted to show the nonlinear relationship between CD34^+^ cell counts and four-point MACEs. We compared patients in the lowest tertile vs. the medium plus the highest tertiles to evaluate the risk of a low CD34^+^ cell count. Association of CD34^+^ cell levels with CVD outcomes and all-cause mortality was evaluated with Cox hazard regression analysis adjusted for several confounding factors such as age, sex, diabetes mellitus, smoking habit, history of CVD, geriatrics nutritional risk index (GNRI), hemoglobin, C-reactive protein, and intact parathyroid hormone (iPTH) all of which were considered clinically important. Competing risk regression analysis was used to assess the independent risk of two failures: CVD death as the event of interest, and non-CVD death as a competing risk. The proportional hazards assumption for covariates was tested using scaled Schoenfeld residuals. All statistical analyses were conducted with STATA 14.2/SE (Stata Corp.2015, College Station, TX, USA). Differences were considered significant when the p-value was <0.05.

### Ethical considerations

This study was conducted based on the guidelines of the Declaration of Helsinki Principles, and written informed consent from all participants was obtained before blood sample collection. The ethics committee of the Nagoya University Graduate School of Medicine approved the study protocol (ID: 2014–0422).

## Results

### Baseline characteristics

Table 1 shows the baseline demographic, clinical, biochemical, and medication data according to the levels of relative CD34^+^ cells. The mean ratio of CD34^+^ cells to WBC was 0.09 (range, 0.01 to 0.35 for all study patients). Patients in the lowest tertile were more likely to be older, have higher prevalence of pCVD, and to smoke than patients in the medium and highest tertiles. However, no statistically significant differences were found regarding male gender, body mass index, dialysis vintage, diabetes mellitus status, or hypertension. The levels of the following biochemical markers were lower among patients in the lowest tertile: calcium × phosphate product (Ca × Pi), iPTH, serum albumin, and GNRI. Although we found no significant differences, hemoglobin and the white blood cell count tended to be low, and HbA1c tended to be high, in the lowest tertile. In contrast, we found no intergroup differences in C-reactive protein, high- or low-density lipoprotein-cholesterol, and the quantity of HD (kt/v = dialyzer clearance of urea x dialysis time/ volume of distribution of urea).

**Table 1:** Baseline characteristics of study participants according to CD34^+^ tertiles.

Circulating CD34^+^ cell levels					
Characteristics	All (n = 215)	Lowest Tertile (n = 71)	Medium Tertile (n = 72)	Highest Tertile (n = 72)	p value
**Demographic and clinical**					
Follow-up (year)	8.0±4.6	6.4±4.6	8.7±4.3	8.8±4.5	0.002*
Male gender [n (%)]	122 (57)	41 (57)	35 (49)	46 (64)	0.178
Age (year)	64.9±11.2	68.0±8.6	64.1±11.5	62.8±12.6	0.016*
Body mass index (kg/m^2^)	20.8±3.2	20.5±2.8	20.9±3.6	21.0±3.2	0.617
Dialysis-vintage (year)	8.0±7.2	7.0±6.8	8.4±7.7	8.7±7.0	0.311
Diabetes [n (%)]	105 (49)	41 (57)	30 (42)	34 (47)	0.150
Hypertension [n (%)]	155 (72)	54 (75)	49 (68)	52 (72)	0.556
History of CVD [n (%)]	94 (44)	37 (52)	32 (44)	25 (35)	0.110
Smoking habit [n (%)]	62 (29)	30 (42)	17 (24)	15 (21)	0.007*
**Biochemical markers**					
Relative CD34^+^ cells	0.09±0.06	0.04±0.01	0.07±0.01	0.15±0.05	< 0.001*
Hemoglobin (g/dl)	10.4±1.2	10.2±1.2	10.4±1.0	10.6±1.4	0.147
WBC (10^3^/μl)	5.9±1.9	5.3±1.7	5.9±1.5	6.6±2.1	0.329
C-reactive protein (mg/dl)	0.45±1.01	0.63±1.05	0.29±0.40	0.43±1.33	0.129
HDL cholesterol (mg/dl)	41.3±13.9	40.3±11.9	43.4±17.2	40.1±11.8	0.271
LDL cholesterol (mg/dl)	76.2.±27.0	78.5±26.7	74.6±28.6	75.5±26.1	0.673
Ca x Pi	59.8±11.9	47.5±12.0	49.0±11.5	52.8±11.7	0.020*
Intact-PTH (ng/ml)	123.1±114.7	95.7±65.5	136.2±150.6	137.0±107.8	0.048*
Kt/V_urea_	1.5±0.2	1.5±0.3	1.4±0.3	1.5±0.2	0.108
HbA1c (%)	6.1±1.6	6.5±2.0	6.2±1.5	5.6±1.1	0.064
Albumin (g/dl)	3.6±0.3	3.5±0.3	3.6±0.3	3.6±0.3	0.013*
GNRI	92.8±7.6	90.9±7.0	93.7±8.0	93.7±7.6	0.044*
**Medications**					
Erythropoietin (u/kg)	96.7±67.8	110.4±63.4	91.1±65.9	88.9±72.5	0.114
Statins [n (%)]	27 (13)	10 (14)	8 (11)	9 (13)	0.866
ACE-inhibitor [n (%)]	37 (17)	14 (19)	9 (13)	14 (19)	0.432
ARB [n (%)]	85 (39)	33 (46)	20 (28)	32 (44)	0.043*
Ca^++^ antagonist [n (%)]	132 (61)	48 (67)	42 (58)	42 (58)	0.424
Β-blocker [n (%)]	45 (21)	21 (29)	17 (24)	7 (10)	0.011*

Data are summarized in mean ± SD or, for binary variables, number (frequency). Tertiles are ordered from lowest relative levels of CD34^+^ cells to highest levels: lowest tertile <0.06; middle tertile ≥0.06 and <0.09; and highest tertile ≥0.09.

*P<0.05: statistically significant association

### Factors associated with the lowest tertile of CD34^+^ cell levels

We examined the ability of certain variables to predict the lowest tertile of relative CD34^+^ cell levels. In univariable analysis, factors that were significantly associated with the lowest tertile were age (odds ratio (OR) 1.04, 95% confidence interval (CI) 1.01–1.07), smoking habit (OR 2.63, 95% CI 1.42–4.86), lower GNRI (OR 0.95, 95% CI 0.91–0.99), lower Ca × Pi (OR 0.98, 95% CI 0.95–0.99), lower iPTH (OR 0.99, 95% CI 0.98–0.99), and erythropoietin (OR 1.01, 95% CI 1.00–1.01). Whereas, in multivariable analysis age and smoking habits were significantly associated with the lowest tertile of CD34^+^ cell levels (Table 2).

**Table 2:** Associated factors with the lowest level of CD34^+^ cells using logistic regression analysis.

	Univariable		Multivariable	
Characteristics	OR (95% CI)	P value	OR (95% CI)	P value
Male gender	1.06 (0.60–1.89)	0.835	0.50 (0.23–1.06)	0.072
Age (year)	1.04 (1.01–1.07)	0.006*	1.04 (1.00–1.08)	0.031*
Smoking habit	2.63 (1.42–4.86)	0.002*	4.77 (2.16–10.57)	< 0.001*
Diabetes	1.71 (0.96–3.03)	0.068	1.49 (0.74–3.01)	0.261
History of CVD	1.66 (0.94–2.95)	0.083	0.87 (0.40–1.89)	0.724
C-reactive protein (mg/dl)	1.29 (0.96–1.74)	0.095	1.14 (0.78–1.66)	0.489
Hemoglobin (g/dl)	0.80 (0.63–1.03)	0.077	0.88 (0.65–1.19)	0.399
GNRI	0.95 (0.91–0.99)	0.014*	0.97 (0.93–1.02)	0.278
CaxPi	0.98 (0.95–0.99)	0.048*	0.99 (0.96–1.02)	0.455
iPTH (ng/ml)	0.99 (0.98–0.99)	0.012*	0.99 (0.99–1.00)	0.184
Erythropoietin (unit/Kg)	1.01 (1.00–1.01)	0.039*	1.00 (0.99–1.01)	0.485
ACE-inhibitor	1.29 (0.62–2.70)	0.494	1.14 (0.50–2.59)	0.758
Angiotensin receptor antagonist	1.54 (0.86–2.74)	0.145	1.47 (0.76–2.84)	0.256
Statins	1.23 (0.53–2.83)	0.636	1.04 (0.39–2.80)	0.940

OR odd ratio

*P<0.05: statistically significant association

### Associations of CD34^+^ cell levels with CV outcomes and all-cause mortality

**Fig 1:**
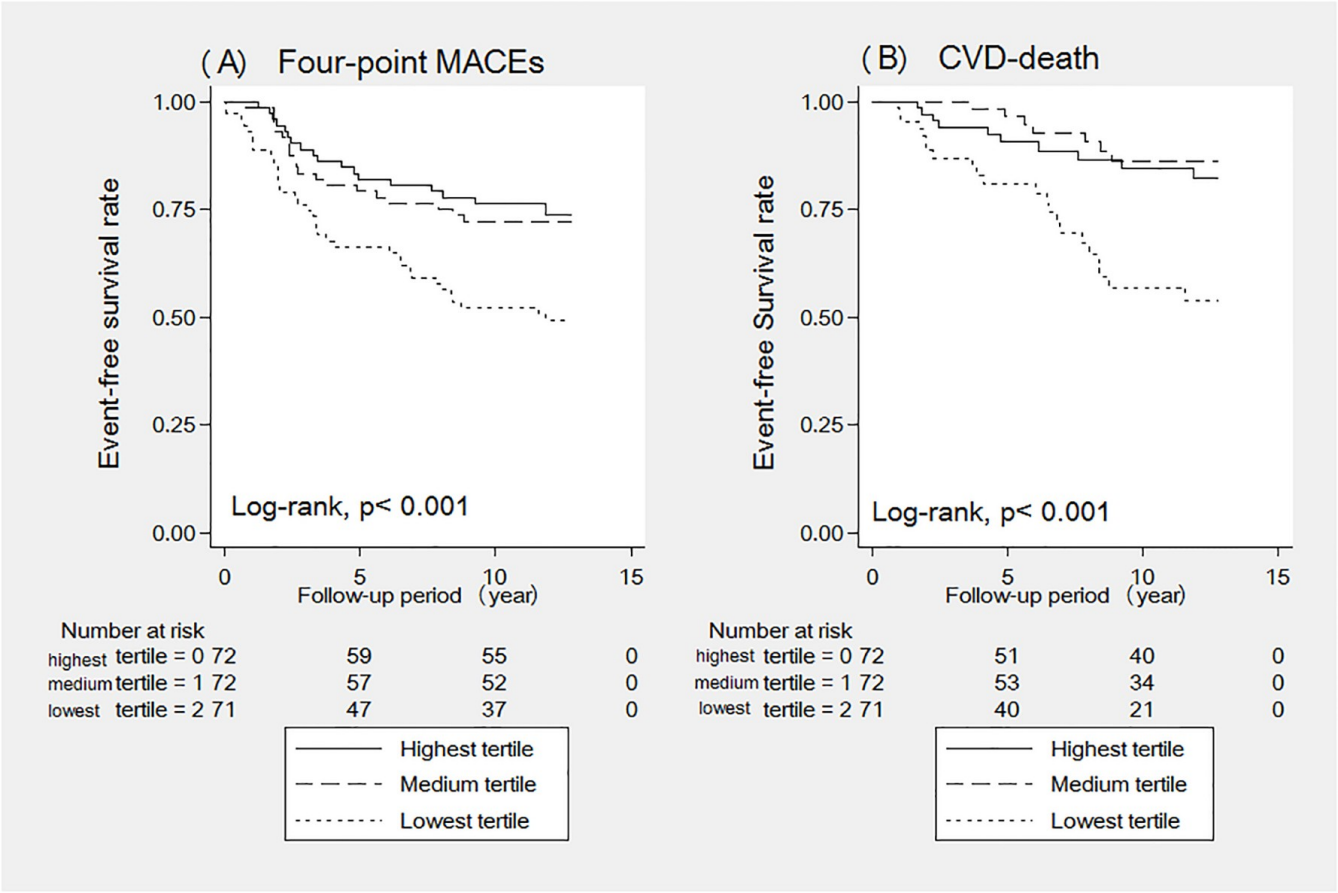
(A and B). Cumulative event-free survival for 4-point MACEs, and CVD-death according to the lowest, medium, and highest relative CD34^+^ cell tertiles.

**Fig 2:**
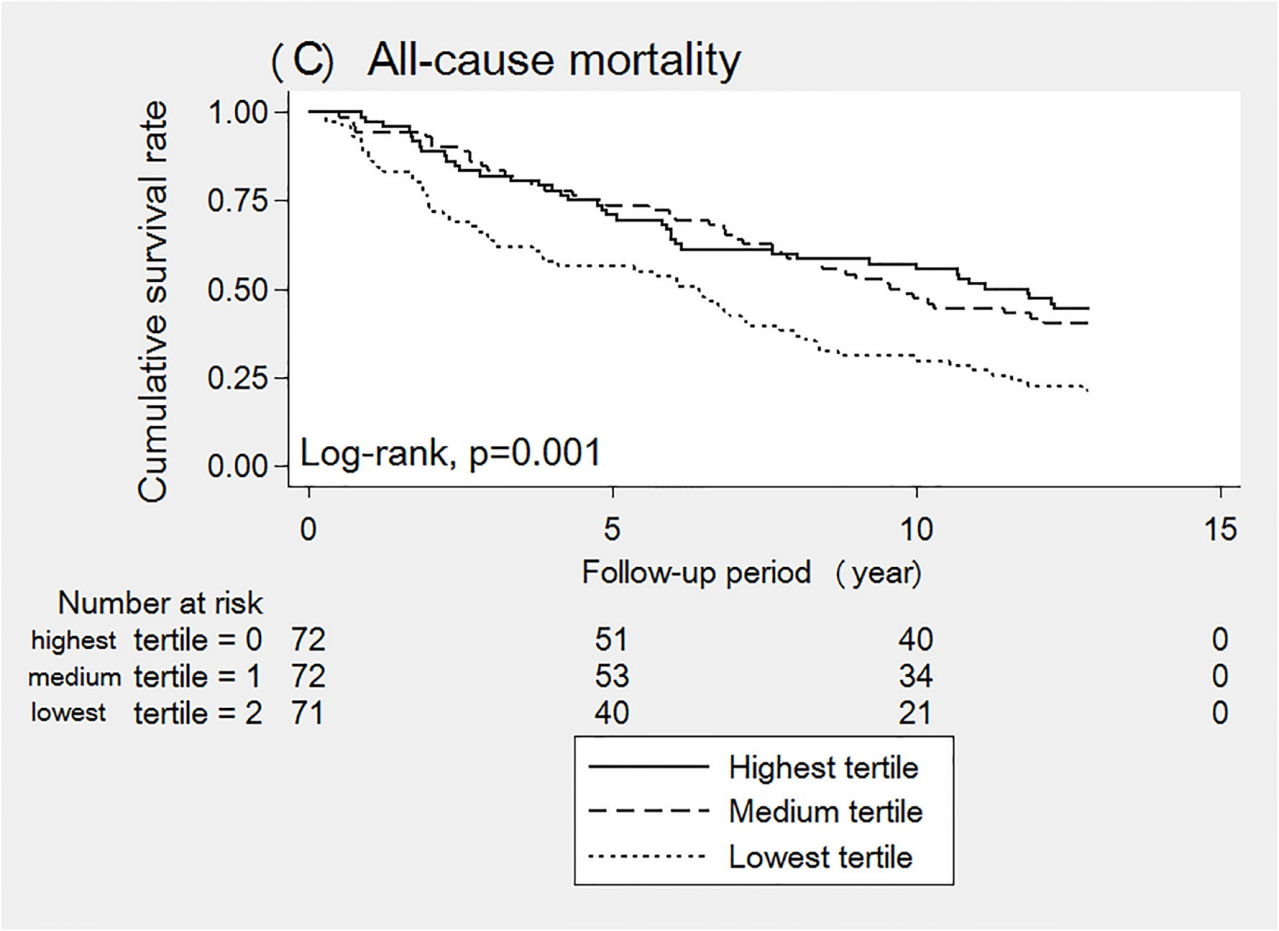
Cumulative survival rate for all-cause mortality according to the lowest, medium, and highest relative CD34^+^ cell tertiles.

**Fig 3:**
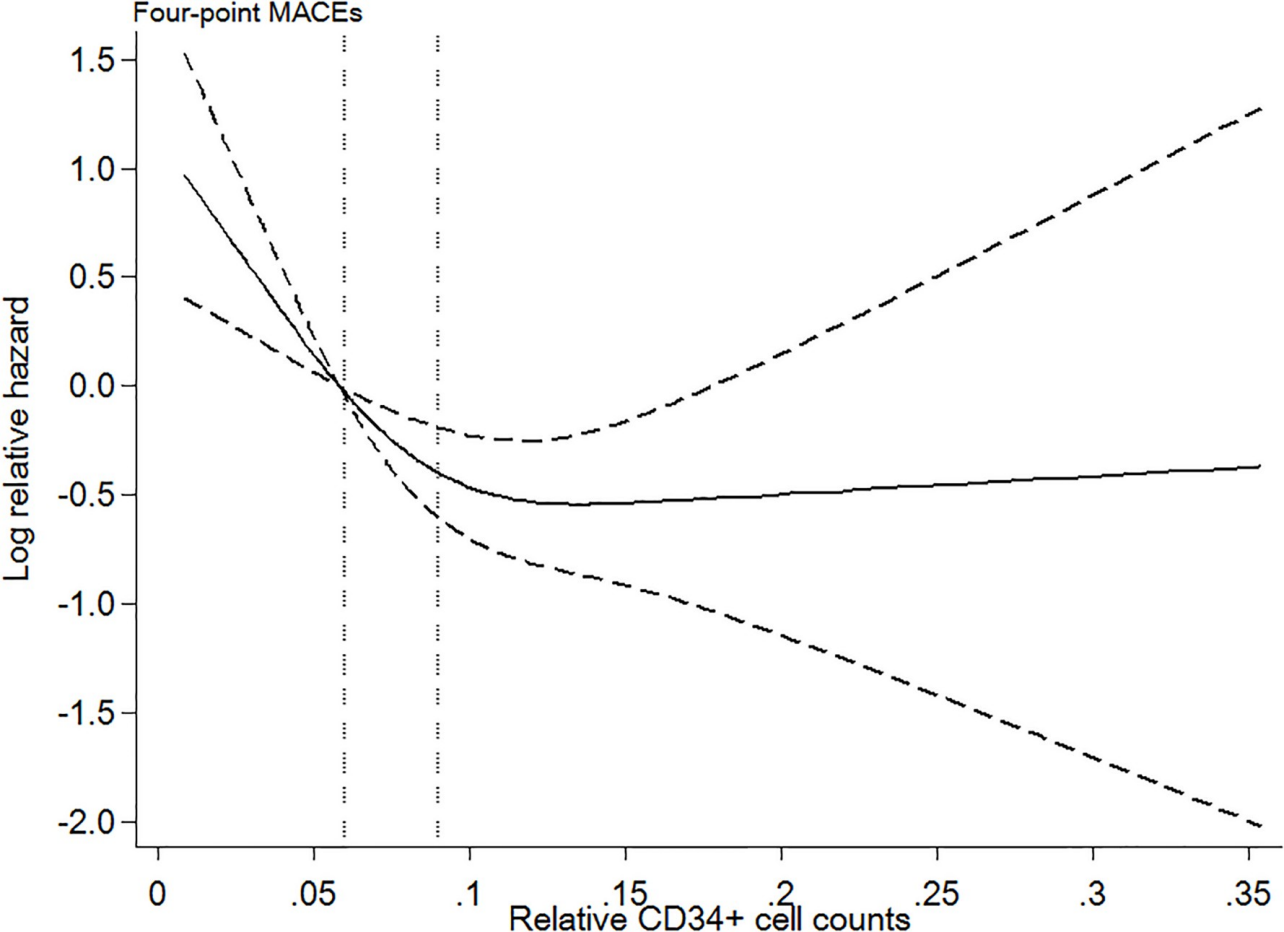
Log relative hazard of 4-point MACEs for relative CD34^+^ cell counts in the restricted cubic spline curve. The reference is the cut-off point between the lowest and medium tertiles; the two vertical dotted lines indicate the cut-off for these tertiles (0.06 and 0.09, respectively). The graph has three knots that are truncated at the 1^st^ and 99^th^ percentiles.

Among 139 (64.7%) patients who died during the mean observation period of 8.0 ± 4.6 years, 39 (28.1%) patients died from CVD, and 100 (71.9%) patients died from non-CVD causes. The crude mortality rate was 8.0 per 100 patient-years. Kaplan-Meier analyses showed a significantly lower survival rate in patients with the lowest level of CD34^+^ cells than in those with the medium and highest levels of CD34^+^ cells (Figs 1 and 2; log-rank p ≤ 0.001). No significant difference was found in the survival rate between the highest and medium tertiles. In addition, the restricted cubic spline curve demonstrated that patients with a lower CD34^+^ cell count had an elevated risk of four-point MACEs (Fig 3).

In unadjusted Cox hazard regression, the lowest tertile, history of CVD, and C-reactive protein were associated with four-point MACEs. In multivariable Cox hazard models, the lowest tertile (HR 1.80, 95% CI 1.08–2.99, p = 0.023) and history of CVD (HR2.63, 95% CI 1.22–4.40, p < 0.001) were independent factors associated with four-point MACEs, even after adjusting for gender, age, diabetes, smoking habit, hemoglobin, GNRI, C-reactive protein, and iPTH. Similarly, in unadjusted Cox hazard regression, the lowest tertile, age, history of CVD, hemoglobin, GNRI, and C-reactive protein were associated with CVD death. In multivariable Cox hazard models, the lowest tertile (HR 2.50, 95% CI 1.23–5.10, p = 0.011), and history of CVD (HR 4.67, 95%CI 2.07–10.52, p < 0.001) were significant predictors of CVD-death (Table 3). The lowest tertile was also significantly associated with all-cause mortality in the univariable Cox hazard model (HR 1.88, 95% CI 1.34–2.64, p < 0.001), but the association was attenuated and not significant when adjusted for above mentioned confounding factors (HR 1.15, 95% CI 0.78–1.68, p = 0.481) (Table 4).

**Table 3:** The hazard ratio of associated factors for cardiovascular outcomes.

	*four-point MACEs*				*CVD-death*			
Characteristics	*Univariable*		*Multivariable*		*Univariable*		*Multivariable*	
	*HR (95% CI)*	*P value*	*HR (95% CI)*	*P value*	*HR (95% CI)*	*P value*	*HR (95% CI)*	*P value*
Lowest relative CD34^+^ tertile vs. medium+highest tertiles	2.24 (1.42–3.52)	0.001*	1.80 (1.08–2.99)	0.023*	3.63 (1.93–6.86)	<0.001*	2.50 (1.23–5.10)	0.011*
Male gender	1.21 (0.76–1.92)	0.424	1.12 (0.65–1.93)	0.683	1.01 (0.54–1.89)	0.979	1.00 (0.47–2.14)	0.991
Age-per 10 years	1.01(0.99–1.03)	0.178	1.00 (0.98–1.03)	0.948	1.39 (1.03–1.87)	0.030*	0.99 (0.96–1.03)	0.748
Dialysis-vintage (year)	1.01 (0.98–1.04)	0.443	-	-	1.02 (0.97–1.06)	0.489	-	-
Diabetes	1.56 (1.99–2.46)	0.058	1.18 (0.71–1.97)	0.526	1.28 (0.69–2.41)	0.437	1.03 (0.50–2.15)	0.930
Hypertension	1.51 (0.87–2.62)	0.144	-	-	1.46 (0.67–3.19)	0.337	-	-
Smoking habit	1.56 (0.97–2.51)	0.068	1.27 (0.72–2.23)	0.411	1.30 (0.66–2.56)	0.452	1.19 (0.53–2.68)	0.673
History of CVD	2.77 (1.73–4.44)	< 0.001*	2.63 (1.22–4.40)	<0.001*	5.95 (2.82–12.55)	<0.001*	4.67 (2.07–10.52)	<0.001*
Hemoglobin (g/dl)	0.91 (0.75–1.10)	0.313	0.99 (0.81–1.21)	0.942	0.69 (0.52–0.91)	0.010*	0.91 (0.67–1.24)	0.557
GNRI	0.99 (0.96–1.02)	0.451	1.01 (0.97–1.05)	0.614	0.92 (0.88–0.97)	0.002*	0.96 (0.90–1.01)	0.125
C-reactive protein (mg/dl)	1.22 (1.03–1.45)	0.021*	1.15 (0.95–1.41)	0.161	1.44 (1.14–1.82)	0.002*	1.30 (0.99–1.69)	0.056
iPTH	1.00 (0.99–1.00)	0.145	1.00 (0.99–1.00)	0.744	0.99 (0.99–1.001)	0.289	1.00 (0.99–1.00)	0.821

MACEs Major adverse cardiovascular disease, HR hazard ratio, CI confidence interval

*p< 0.05: statistically significant association

**Table 4:** Hazard ratio of associated factors for all-cause mortality.

Characteristics	*Univariable*		*Multivariable*	
	HR (95% CI)	P value	HR (95% CI)	P value
Lowest relative CD34^+^ tertile vs. medium+highest tertiles	1.88 (1.34–2.64)	< 0.001*	1.15 (0.78–1.68)	0.481
Male gender	1.42 (1.00–1.99)	0.047*	1.39 (0.92–2.08)	0.115
Age-per 10 years	1.58 (1.35–1.85)	< 0.001*	1.03 (1.01–1.05)	< 0.001*
Dialysis-vintage (year)	1.01 (0.98–1.03)	0.550	-	-
Diabetes	1.36 (0.97–1.90)	0.072	1.13 (0.77–1.64)	0.531
Hypertension	1.05 (0.72–1.53)	0.805	-	-
Smoking habit	1.35 (0.95–1.94)	0.099	1.45 (0.95–2.23)	0.087
History of CVD	1.98 (1.42–2.77)	< 0.001*	1.33 (0.92–1.92)	0.125
Hemoglobin (g/dl)	0.79 (0.68–0.91)	0.002*	0.93 (0.79–1.09)	0.376
GNRI	0.92 (0.90–0.95)	< 0.001*	0.94 (0.92–0.97)	< 0.001*
C-reactive protein (mg/dl)	1.35 (1.18–1.54)	< 0.001*	1.17 (1.02–1.35)	0.028*
iPTH (ng/dl)	0.99 (0.99–0.99)	0.038*	0.99 (0.99–1.00)	0.539

HR hazard ratio, CI confidence interval

*p<0.05: statistically significant association

Using the Fine and Gray competing regression model, treating non-CVD death as a competing risk, the results showed a significantly higher Sub-hazard ratio (SHR) of CVD death in patients in the lowest tertile compared to those in the medium and highest tertiles (SHR 2.68, 95% CI 1.30–5.52, p = 0.008) (Table 5).

**Table 5:** Hazard and Sub-hazard ratio of CVD-death in the lowest tertile of relative CD34^+^ cells by Cox regression and competing regression analysis.

Multivariable models	HR (95% CI)	P value	SHR (95% CI)	P value
Lowest CD34^+^ tertile vs. medium+highest tertiles	2.50 (1.23–5.10)	0.011*	2.68 (1.30–5.52)	0.008*

HR hazard ratio, SHR sub hazard ratio, CI confidence interval, CD34^+^ levels were adjusted for male gender, age, diabetes, smoking, pCVD, hemoglobin, GNRI, C-reactive protein, and iPTH.

*P<0.05: statistically significant association

## Discussion

This study shows that reduced levels of CD34^+^ cells predict CVD outcomes defined as four-point MACEs and CVD death in a cohort of 215 patients on maintenance HD over an average of 8.0 years of follow-up. The HRs of four-point MACEs and CVD death for the lowest vs. medium plus highest tertiles were 1.80 and 2.50, respectively, after accounting for a number of potential confounders. In our study, reduced levels of CD34^+^ cell counts were not significantly associated with all-cause mortality in the adjusted model. Age and smoking habit showed a significant association with low CD34^+^ cell counts. To the best of our knowledge, this investigation utilized the longest follow-up period in patients on maintenance HD. In our study, more than 50% of four-point MACEs and CVD deaths occurred after 5.3 years of follow-up, which supports the need to implement a longer-term observational study.

Several factors affect the number of EPCs, such as age, smoking, type 2 diabetes, and CVD[19]. Indeed, our study showed similar results for these factors. The inverse significant association between levels of CD34^+^ cell with age and smoking in HD patients may be due to persistent endothelial injury and exhaustion of migration of progenitor cells in the bone marrow that lead to an eventual depletion in the CD34^+^ cell count[20,21]. However, the mean number of absolute CD34^+^ cell in HD patients in this cohort was 0.49 ± 0.32 cells(/μl) as shown in our previous paper[18], which was much lower compared to that in non-HD patients without DM (mean CD34^+^ cell count 1.2 ± 0.1 cells (/μl) as reported in a previous study[22]. These severely low CD34^+^ cell counts are consistent with a previous study that investigated patients undergoing maintenance HD[17]. Importantly, uremic toxins are considered to be a cause of the decline in circulating CD34^+^ cells[23]. Therefore, low CD34^+^ cell counts are considered a characteristic of patients on maintenance HD therapy and maybe a meaningful predictive factor. Some drugs such as angiotensin II receptor antagonist, statin and erythropoietin have been shown to affect the number of circulating CD34^+^ cell[24,25]. However, there was no favorable effect of these drugs on the levels of CD34^+^ cells in our cohort, probably because chronic HD patients had low capacity to produce CD34^+^ cells.

The Kaplan-Meier curves clearly showed patients with lowest tertile has significantly low survival rate than those in the medium and highest tertiles, but no significant difference was found in the survival rate between medium and highest tertile. In addition, no cut-off has been established for CD34^+^ cell counts relative to white blood cells to determine a high risk of CVD outcomes. Especially in patients receiving maintenance HD therapy, we need to carefully interpret the value because of the gap reported between patients who are and who are not receiving HD therapy. In our current study, we evaluated the association between CD34^+^ cell counts and four-point MACEs using a restricted cubic spline curve and found a dose-response association with an intensively increased risk in patients with low CD34^+^ cell counts. These findings supported our comparison of the lowest tertile of CD34^+^ cells with the others (medium and highest tertile) with the cut-off of 0.06.

We need to discuss why reduced levels of CD34^+^ cells predict CVD outcomes. Increasing evidence documents that circulating EPCs including CD34^+^ cells play a potential role in maintenance of endothelial integrity, function, and postnatal neovascularization[26–28]. However, the detailed mechanism is still unclear regarding whether low numbers of EPCs directly cause CVD events or are associated with chronic inflammation, hematopoietic exhaustion, and bone marrow abnormalities which lead to the occurrence of CVD[29] Basically, patients receiving HD therapy have 20 times elevated risk of CVD outcomes than in the general population[30]. Because traditional CVD risk factors, such as gender, age, smoking, diabetes, and CKD-related risk factors (i.e. anemia, chronic kidney disease-mineral bone disorder, GNRI; an index for assessing nutritional status, C-reactive protein; a marker of inflammation) are common in this population[30], we performed multivariate analyses adjusting the level of CD34^+^ cells with all these variables. We found that level of relative CD34^+^ cells and history of CVD were independent risk factors for future CVD outcomes. Therefore, we hypothesized that levels of CD34^+^ cell counts have a causal effect for CVD outcomes. Further studies are needed to elucidate the exact protective mechanism of CD34^+^ cells in chroninc HD patients. In contrast, low levels of CD34^+^ cells did not predict all-cause mortality in this study. We do not have information about the specific causes of non-CVD deaths that will be needed to assess the details of this observation. Nonetheless, these findings were consistent with previous studies[12,13,17].

Regarding cell surface markers, we measured only CD34 for three reasons: quantification method with single antibody staining for CD34^+^ cells is easy, the results are highly reproducible [18,31], and among EPCs phenotypes (CD34^+^, CD133^+^, KDR^+^) the subset of CD34^+^ circulating cells showed good correlation with cardiovascular parameter[31]. Furthermore, it is reported that the administration of CD34^+^ cells after stroke improves neurogenesis through angiogenesis in a mouse model[32]. Also, the circulating CD34^+^ cell count is noteworthy because accumulating evidence[15,22,31,33] supports CD34^+^ cells use in cell therapy for cardiac and limb ischemia in human[34,35].

In this paper, we used the levels of relative CD34^+^ cells rather than level of absolute CD34^+^ cells among patients on HD because circulating CD34^+^ cell count in per unit of volum (μl), can be artificially affected by hemodilution and hemoconcentration, which are more common among HD patients[13]. We also analyzed the effect of lowest vs. medium plus highest levels of absolute CD34^+^ cell and the absolute number of CD34^+^ cells, as a continouos value, in association with CVD outcomes and all-cuase mortality. We did not find any significant association. The data are shown in the S1 Table. Therefore, we concluded that the ratio of CD34 to white blood cells in patients subjected to day-to-day changes in body fluids reflects a more accurate and an independent predictive factor for CVD outcomes in HD patients.

One of the strong points of the present study compared to previous reports are the longest follow-up period of up to 12.8 years. We found that the relative CD34^+^ cell count was an independent predictive factor of four-point MACEs and CVD death in patients on maintenance HD. In addition, to reduce the bias generated by non-CVD deaths, we analyzed the SHR of CVD death vs. non-CVD death as a competing risk. Again, the resluts demonstrated that a low level of relative CD34^+^ cells was strongly related to future CVD deaths.

We acknowledge limitations of this study. First, we do not have detailed information about non-CVD death that will be needed to explore the reason why we did not observe an association between relative CD34^+^ cell counts and all-cause mortality. Second, this study does not have the anatomic data such as coronary calcium scoring or plaque burden on coronary CT angiography or ultrasound of atherosclerotic changes of carotid arteries. Third, this study had a small sample size and was conducted in a single health center.

In summary, a low level of CD34^+^ cells in the circulation predicts long-term CVD outcomes among patients on maintenance HD. Further studies are needed to assess whether interventions such as ceasing smoking will increase CD34^+^ cell number and improve patient outcomes.

## Supporting information

S1 Table This table shows hazard ratio of lowest CD34^+^ cells level and continuous value of CD34^+^ cells for CVD outcomes and all-cause mortality. (DOCX)
