# Supplementary material for: Circulating levels of CD34+ cells predict long-term cardiovascular outcomes in patients on maintenance hemodialysis
Source: PLoS One. 2019 Oct 4;14(10):e0223390. doi: 10.1371/journal.pone.0223390 (PMC6777758; doi:10.1371/journal.pone.0223390)
Supplement: S1 Table — (DOCX) [file pone.0223390.s001.docx]

| Cell counting (absolute CD34^+^) | Four-point MACEs | CVD-death | All-cause mortality |
| --- | --- | --- | --- |
| Lowest vs. medium+highest tertile  P | 1.47 (0.89-2.43)  0.132 | 1.63 (0.83-3.19)  0.158 | 0.97 (0.67-1.41)  0.867 |
| Continuous value of CD34^+^ cells (/µl)  P | 0.71 (0.29-1.71)  0.441 | 0.43 (0.12-1.57)  0.200 | 0.88 (0.49-1.55)  0.649 |

**S1 Table. The hazard ratio of lowest CD34^+^ cells level and continuous value of CD34^+^ cells for CVD outcomes and all-cause mortality**

The hazard ratios of absolute CD34^+^ cell adjusted for male gender, age, diabetes, smoking, pCVD, hemoglobin, GNRI, C-reactive protein, and iPTH.
